# Evaluating the correlation of sclerostin levels with obesity and type 2 diabetes in a multiethnic population living in Kuwait

**DOI:** 10.3389/fendo.2024.1392675

**Published:** 2024-04-22

**Authors:** Tahani Alramah, Preethi Cherian, Irina Al-Khairi, Mohamed Abu-Farha, Thangavel Alphonse Thanaraj, Ahmed N. Albatineh, Fayez Safadi, Hamad Ali, Muhammad Abdul-Ghani, Jaakko Tuomilehto, Heikki A. Koistinen, Fahd Al-Mulla, Jehad Abubaker

**Affiliations:** ^1^ Department of Biochemistry and Molecular Biology, Dasman Diabetes Institute, Dasman, Kuwait; ^2^ Department of Translational Research, Dasman Diabetes Institute, Dasman, Kuwait; ^3^ Genetics and Bioinformatics Department, Dasman Diabetes Institute, Dasman, Kuwait; ^4^ Faculty of Medicine, Kuwait University, Kuwait City, Kuwait; ^5^ Department of Anatomy and Neurobiology, Northeast Ohio Medical University, Rootstown, OH, United States; ^6^ Rebecca D. Considine Research Institute, Akron Children Hospital, Akron, OH, United States; ^7^ Department of Medical Laboratory Sciences, Faculty of Allied Health Sciences, Health Sciences Center, Kuwait University, Jabriya, Kuwait; ^8^ Division of Diabetes, University of Texas Health Science Center, San Antonio, TX, United States; ^9^ Department of Public Health and Welfare, Finnish Institute for Health and Welfare, Helsinki, Finland; ^10^ Department of Public Health, University of Helsinki, Helsinki, Finland; ^11^ Saudi Diabetes Research Group, King Abdulaziz University, Jeddah, Saudi Arabia; ^12^ Internal Medicine and Endocrinology, Minerva Foundation Institute for Medical Research, Helsinki, Finland; ^13^ Department of Medicine, University of Helsinki and Helsinki University Hospital, Helsinki, Finland

**Keywords:** SOST, gender, T2DM, obesity, ethnicity, bone metabolism

## Abstract

Obesity and Type 2 Diabetes Mellitus (T2DM) are intricate metabolic disorders with a multifactorial etiology, often leading to a spectrum of complications. Recent research has highlighted the impact of these conditions on bone health, with a particular focus on the role of sclerostin (SOST), a protein molecule integral to bone metabolism. Elevated circulating levels of SOST have been observed in patients with T2DM compared to healthy individuals. This study aims to examine the circulating levels of SOST in a multiethnic population living in Kuwait and to elucidate the relationship between SOST levels, obesity, T2DM, and ethnic background. The study is a cross-sectional analysis of a large cohort of 2083 individuals living in Kuwait. The plasma level of SOST was measured using a bone panel multiplex assay. The study found a significant increase in SOST levels in individuals with T2DM (1008.3 pg/mL, IQR-648) compared to non-diabetic individuals (710.6 pg/mL, IQR-479). There was a significant gender difference in median SOST levels, with males exhibiting higher levels than females across various covariates (diabetes, IR, age, weight, and ethnicity). Notably, SOST levels varied significantly with ethnicity: Arabs (677.4 pg/mL, IQR-481.7), South Asians (914.6 pg/mL, IQR-515), and Southeast Asians (695.2 pg/mL, IQR-436.8). Furthermore, SOST levels showed a significant positive correlation with gender, age, waist circumference, systolic and diastolic blood pressure, fasting blood glucose, HbA1c, insulin, total cholesterol, triglycerides, HDL, LDL, ALT, and AST (p-Value ≥0.05). South Asian participants, who exhibited the highest SOST levels, demonstrated the most pronounced associations, even after adjusting for age, gender, BMI, and diabetes status (p-Value ≥0.05). The observed correlations of SOST with various clinical parameters suggest its significant role in the diabetic milieu, particularly pronounced in the South Asian population compared to other ethnic groups.

## 1 Introduction

Obesity and diabetes are known metabolic diseases that result due to disturbance in the lipid and glucose metabolism leading to imbalance in energy homeostasis (1, 2). The prevalence of obesity has been continuously increasing in most countries since 1980, as per the report by the Global Burden of Disease Group in 2017 (3). Obesity is defined by a body mass index (BMI) of more than or equal to 30 kg/m2, which occurs due to the imbalance between energy intake and expenditure, resulting in excess accumulation of body fat (WHO and CDC). Another metabolic disease characterized by hyperglycemia and is increasing in prevalence is diabetes mellitus (DM), particularly T2DM, which accounts for 90–95% of all patients with diabetes and is expected to increase to 439 million by 2030 (4, 5). Both obesity and T2DM are complex metabolic disorders of multifaceted etiology accompanied by a series of complications, including macrovascular diseases (hypertension, hyperlipidemia, heart attacks, coronary artery disease, strokes, cerebral vascular disease, and peripheral vascular disease), microvascular diseases (retinopathy, nephropathy, and neuropathy), and cancers (6). Recently, studies have shown that bone health is also compromised in people with obesity and T2DM (7).

The skeletal system is highly dynamic and constantly undergoes repair and remodeling (8). Normal bone remodeling is necessary for fracture healing and skeleton adaptation to mechanical use. A disruption in the equilibrium between bone formation and resorption processes can result in bone-related diseases such as osteoporosis (9). Recently, bone has been recognized as an endocrine organ capable of synthesizing and secreting a variety of bioactive compounds that regulate bone remodeling and affect the metabolic processes throughout the body (9, 10). The endocrine function of bone is mediated through the secretion of several hormones, such as osteocalcin (OC), fibroblast growth factor 23 (FGF23), lipocalin 2 (Lcn2), and sclerostin (SOST) (11, 12).

SOST is a glycoprotein with a molecular weight of̴27 kDa, which is crucial in regulating bone metabolism (13). Encoded by the SOST gene, it is predominantly synthesized by osteocytes and is vital in maintaining bone homeostasis (14). SOST influences both bone formation and remodeling processes by acting as an inhibitory agent in the Wnt/β-catenin signaling pathway via binding to LDL receptor-related proteins 5/6 (LRP5/6) thereby negatively regulating bone formation (15, 16). Mutations in the SOST gene, leading to a deficiency of SOST, can give rise to two uncommon autosomal recessive bone-hardening conditions: Sclerosteosis and van Buchem disease (17).

Circulating SOST levels have been found to be altered in individuals with T2DM (18, 19). Patients with T2DM show higher circulating levels of SOST compared with healthy individuals. Since SOST is mainly produced by osteocytes this indicates that fluctuations in glucose levels could significantly impact the primary cells responsible for bone health. Specifically, when blood glucose levels vary considerably from the standard 80–140 mg/dL range it may negatively affect osteocytes (20). This is inferred from the observation that T2DM patients often display disrupted bone metabolism, making them more susceptible to fractures and other bone-related issues (12, 21). Notably, the risk of fractures in T2DM patients is nearly double that of healthy individuals, even if their bone mineral density (BMD) is normal or elevated (11). This points to potential issues with bone quality, possibly affecting its strength or micro-architecture. Moreover, Daniele et al. reported an increase in blood SOST levels, which was significantly associated with insulin resistance in skeletal muscle, liver, and adipose tissue in patients with diabetes (22). Additionally, in a study done on Indian male individuals who have been diagnosed with T2DM, the authors observed a notable increase in the levels of circulating SOST when compared to the healthy male control group (23). This finding was consistent with the expression levels of SOST mRNA. Furthermore, in a study done on SOST knockout mice the author showed that these mice exhibit increased bone mass, decreased fat mass, and increased insulin sensitivity (24). The authors also demonstrated that treatment with anti-SOST antibodies decreased fat mass in wild-type mice and enhanced differentiation of adipocytes.

The role of SOST in obesity and diabetes is well documented. We hypothesize that Individuals with obesity and T2DM will have higher circulating levels of SOST compared to healthy individuals. Additionally, we predict that ethnic variations within the population residing in Kuwait may influence SOST plasma levels due to the high prevalence of obesity and T2DM. This study aims to investigate the circulating level of SOST in the population living in Kuwait. Also, since the Kuwaiti population is comprised of people from different ethnic backgrounds, the impact of ethnic variation in relationships between SOST and obesity/diabetes was explored. Furthermore, the objective of this study is to identify the factors that may be associated with SOST levels and their possible health implications.

## 2 Materials and methods

### 2.1 Participants and the study design

Kuwait Diabetes Epidemiological Program (KDEP) was a cross-sectional, population-based study conducted between 2011 and 2014 at the Dasman Diabetes Institute (DDI). The study protocol was approved by the Ethics Review Committee at DDI under the reference RA2011-003, and done in accordance with the principles of the Declaration of Helsinki and good clinical practice guidelines. Participants were recruited using a stratified random sampling method designed to ensure a balanced representation across all seven governorates of Kuwait. The primary criteria for exclusion from the study were refusal to sign the consent form, being younger than 21, or suffering from an ongoing infection. This method of participant selection aimed to ensure a diverse and representative sample of the adult population as previously described (25–27).

### 2.2 Anthropometric and vital signs measurements

Anthropometric measurements were recorded, including body weight, height, and waist circumference (WC). Height and weight measurements were conducted using calibrated scales and fixed height bars while participants wore light clothing and were barefoot. The waist circumference was measured using a tension tape at the iliac crest and mid-axillary line after a normal exhalation with relaxed arms. BMI was calculated using the standard formula: weight in kilograms divided by height in meters squared. Blood pressure was measured with an Omron HEM-907XL Digital sphygmomanometer, taking three separate readings with 5–10 minute rests between them and the final values were averaged from these readings.

### 2.3 Laboratory measurements

Blood samples were collected from each participant after an overnight fasting. The samples were used to measure fasting plasma glucose (FPG), hemoglobin A1c (HbA1c), fasting insulin, triglycerides (TG), total cholesterol (TC), low-density lipoprotein (LDL), and high-density lipoprotein (HDL). The Siemens Dimension RXL chemistry analyzer (Diamond Diagnostics, Holliston, MA) was used for all measurements except for HbA1c, which was measured using the VariantTM device (BioRad, Hercules, CA).

The insulin levels were quantified using the Access Insulin Assay (Beckman Coulter, Brea, CA). The Homeostatic Model Assessment for Insulin Resistance (HOMA-IR) was used to calculate insulin resistance using the following formula: (FBG in mmol/L) × (fasting insulin in mU/L)/22.5.

SOST plasma levels were measured using an R&D custom multiplexing assay following the kit instructions for the Luminex custom-made panel (cat #LXSAHM, R&D, CA, USA). Plasma levels of inflammatory markers were assessed with the multiplexing immunobead array using the Data analyzed with Bio-Plex manager software version 6 (Bio-Rad, Hercules, CA), and the results were calculated using a 5-PL nonlinear standard curve setting in the Bio-Plex manager software version 6.0. Intra-assay coefficients of variation ranged from 1.2% to 3.8%, whereas inter-assay coefficients ranged from 6.8% to 10.2%. The sensitivity of the Luminex assay for SOST is 7.0 pg.ml as per R&D technical specifications, while SOST antibody used for these assays show< 0.5% cross-reactivity with available related molecules and < 50% cross-species reactivity observed with tested species.

### 2.4 Statistical analysis

The analysis was performed on data from a total of 2,083 subjects. Descriptive statistics on population characteristics were carried out and presented as medians (interquartile ranges: IQR). A Pearson chi-squared test, a Fisher’s exact test, and a Wilcoxon rank sum test were used to determine whether there were significant differences between groups. The association between various clinical parameters and SOST was assessed using Spearman’s correlation. Furthermore, subjects were grouped according to the tertiles (two points that divide an ordered distribution into three parts) of markers of interest. Statistical significance was determined by a p-value of 0.05. All statistical analyses were performed using R statistical software (R Core Team, 2020).

## 3 Results

### 3.1 Descriptive analysis of the population

**Figure 1:**
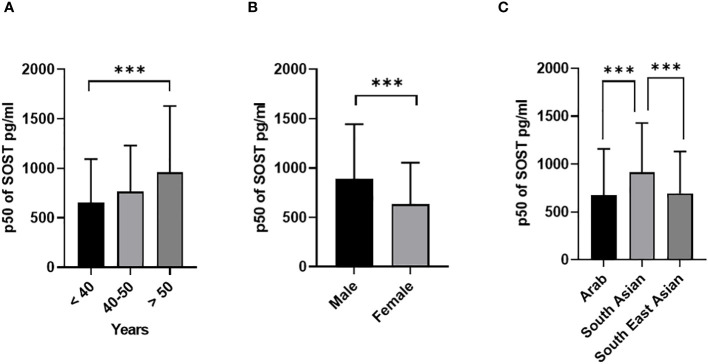
Sclerostin (SOST) level in plasma in all population (n = 2083). The population was stratified based on gender (Male and Female) **(A)**, Age (>40, 40-50, and > 50 years) **(B)**, and Ethnicity (Arab, South Asian, and Southeast Asian) **(C)**. The level of SOST in plasma was determined using a multiplex bone panel. Statistical assessment was 2-sided and considered statistically significant at ***p < 0.001.

**Figure 2:**
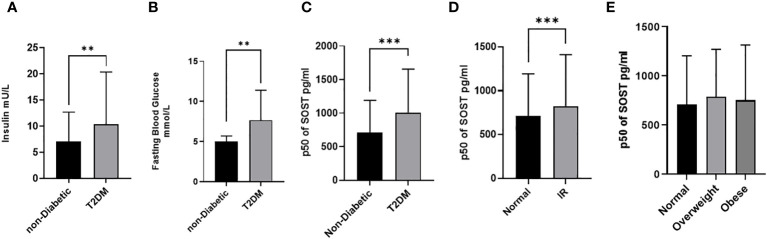
Sclerostin (SOST) level in plasma in all population (n = 2083). The population was stratified based on insulin level **(A)**, Fasting blood glucose (Non-diabetic and T2DM) **(B)**, diabetic status **(C)**, Insulin resistance (HOMA score: ≤ 2 (Normal) and > 2 (IR)) **(D)**, and BMI (BMI: > 24.99 (Normal), 25-29.9 (Overweight), ≥ 30 (Obese)) **(E)**. The level of SOST in plasma was determined using a multiplex bone panel. Statistical assessment was 2-sided and considered statistically significant at **p < 0.001.

Descriptive statistics revealed that the majority of the cohort were male (54.7% **Table 1**), non-Kuwaiti (70.2%), with a median age of 45 years (Interquartile Range, IQR=16), and 36% of the participants were under the age of 40. In terms of ethnicity, 46.6% of the participants were of Arab descent. With respect to the health status, 30.8% of the sample were diagnosed with diabetes, 40.2% were classified as overweight, and 38.7% fell into the obese category. The distributions of the main outcome SOST across several covariates are presented in (**Figures 1**, **2**). Results indicated that males have significantly higher median SOST compared to females (889.3 (554.6), and 634.8 (418.4), pg/ml (IQR) respectively) (**Figure 1A**). There was a significant difference in median SOST across age groups with a dose-response relationship [age: >40 = 655.0 (438.8), 40-50 = 765.5 (464.5), and > 50 = 959.9 (669.1), pg/ml (IQR)] (**Figure 1B**). There were significant differences in median SOST across ethnicity with the South Asians having the highest median SOST [Ethnicity: Arab = 677.4 (481.7), South Asian = 914.6 (515.0), and South East Asian = 695.2 (436.8) pg/ml (IQR)] (**Figure 1C**).

**Table 1:** Demographic characteristics of 2,083 participants.

Characteristics	(%) or median (IQR)
**Gender, n (%) ** Male Female	1161 (55.7%) 922 (44.3%)
**Age, n(%) ** < 40 40-50 > 50	750 (36.0%) 680 (32.7%) 653 (31.3%)
**Ethnicity, n(%) ** Arab South Asian Southeast Asian	** ** 899 (46.6%) 666 (34.5%) 364 (18.9%)
**Diabetes status, n (%) ** Non-Diabetic Diabetic	** ** 1425 (69.2%) 633 (30.8%)
**BMI, n (%) ** Normal BMI Overweight Obese	** ** 441 (21.2%) 837 (40.2%) 805 (38.6)%
**HOMA-IR, n (%) ** HOMA-IR ≤ 2 HOMA-IR > 2	** ** 969 (50.3%) 958 (49.7%)
Hip Circumference, median (IQR)	102.3 (13)
Waist Circumference, median (IQR)	95 (15)
SBP, median (IQR)	131 (26)
DBP, median (IQR)	80 (16)
FBG, median (IQR)	5.3 (1.7)
Insulin, median (IQR)	7.9 (6.7)
TSH, median (IQR)	1.53 (1.14)
FT4, median (IQR)	11.78 (3.43)
FT3, median (IQR)	4.76 (0.78)
HbA1c, median (IQR)	5.8 (1.2)
TC, median IQR)	5.1 (1.33)
AST, median (IQR)	21 (8)
CRP, median (IQR)	3 (2)

IQR, interquartile range; n, number; BMI, body mass index; HOMA-IR, Homeostatic Model Assessment for Insulin Resistance; SBP, systolic blood pressure; DBP, diastolic blood pressure; FBG, fasting blood Glucose; TSH, thyroid stimulating hormone; FT4, free Thyroxine; FT3, free triiodothyronine; HbA1c, glycated haemoglobin; TC, total cholesterol; AST, aspartate aminotransferase; CRP, c-reactive protein.

T2DM patients have significantly higher median SOST [1008.3 (648), pg/ml (IQR)) compared to non-diabetic (710.6 (479) pg/ml (IQR)] with p < 0.001 (**Figure 2A**). Similarly, a significant difference was observed with increased insulin resistance [HOMA-IR score: Normal ≤ 2 = 714.7 (478), and IR > 2 = 825.0 (585) pg/ml (IQR)] (**Figure 2B**). Finally, no significant difference was observed in median level of SOST across the different BMI categories [BMI: > 24.99 (Normal) = 710.1 (495.0), 25-29.9 (Overweight) = 786.5 (484), ≥ 30 (Obese) = 752.3 (563.0), pg/ml (IQR)].

### 3.2 Gender based analysis of the level of SOST

To understand the impact of gender on the circulating level of SOST various parameters were examined stratifying them into male and female categories.

**Figure 3:**
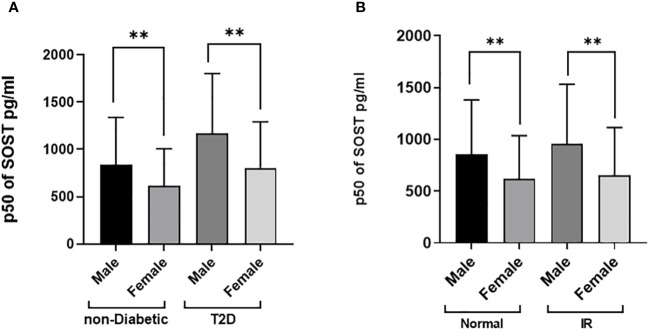
Sclerostin (SOST) level in plasma in the population stratified based on gender (Male and Female). **(A)** Diabetes Status (Non-diabetic and T2DM) **(B)** Insulin resistance [HOMA score: ≤ 2 (Normal) and > 2 (IR)]. The level of SOST in plasma was determined using a multiplex bone panel. Statistical assessment was 2-sided and considered statistically significant at **p < 0.001.

**Figure 4:**
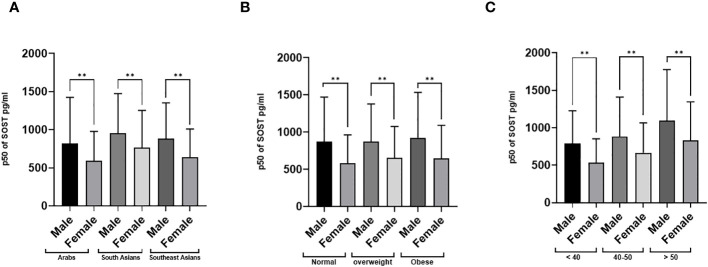
Sclerostin (SOST) level in plasma in the population stratified based on gender (Male and Female). **(A)** Ethnicity (Arab, South Asian, and Southeast Asian) **(B)** BMI [BMI: > 24.99 (Normal), 25-29.9 (Overweight), ≥ 30 (Obese)] **(C)** Age (>40, 40-50, and > 50 years). The level of SOST in plasma was determined using a multiplex bone panel. Statistical assessment was 2-sided and considered statistically significant at **p < 0.001.

The distributions of the main outcome SOST across several covariates are presented in **Figures 3**, **4**. Results indicated that Males have significantly higher median SOST compared to females in both non-diabetic and T2D groups (non-diabetic: Male 833.5 (503.9), and Female 616.4 (390), pg/ml (IQR), in T2D: Male 1168.4 (636.1) and Female 805.8 (487.3), pg/ml) (**Figure 3A**). There was a significant difference in median SOST in both the normal and IR groups (Normal: Male 853.4 (527.4), and Female 622.4 (413.4), pg/ml (IQR), in IR: Male 953.4 (578.4) and Female 656.2 (458), pg/ml) (**Figure 3B**). There were significant differences in median SOST across ethnicity with the South Asians having the highest median SOST [Arab: Male 817.2 (607.2), and Female 591.3 (385), pg/ml (IQR) South Asian: Male 953.4 (519.6), and Female 765.9 (486.4), pg/ml (IQR) and South East Asian: Male 881.9 (469.8), and Female 635.2 (373.4), pg/ml (IQR)] (**Figure 4A**). When the data was categorized on the basis of BMI significant difference was observed in median level of SOST between male and female [Normal: Male 874.5 (598.0), and Female 578.1 (386), pg/ml (IQR). Overweight: Male 868.5 (510), and Female 651.4 (424.9), pg/ml (IQR). Obese: Male 925.2 (608.7), and Female 648.1 (442.8), pg/ml (IQR)] (**Figure 4B**). Finally, when the male and female were further categorized by age the results again showed significant difference among male when compared to female (Age < 40: Male 792.3 (434.8), and Female 532.1 (321.1), pg/ml (IQR). Age 40-50: Male 883.6 (526.7), and Female 666.3 (398.7), pg/ml (IQR). Age 50 <: Male 1093.5 (683.9), and Female 828.8 (518.3), pg/ml (IQR) (**Figure 4C**).

### 3.3 Correlation of SOST level with various clinical parameters

The correlation analysis was performed on all population as presented in **Table 2**. The results show significant positive correlation between SOST level and gender (Male), age, waist circumference, SBP, DBP, insulin, TG, TC, LDL, ALT, AST, FBG, HbA1c,. A significant negative correlation was observed with HDL.

**Table 2:** Correlations between SOST and other covariates and metabolic traits (N=2,083).

Covariate	SOST	Covariate	SOST
Gender	0.256^a^ (<0.001)	Insulin	0.097^b^ (<0.001)
Age	0.300^b^ (<0.001)	TC	0.092^b^ (<0.001)
BMI	0.037^b^ (0.125)	TG	0.152^b^ (<0.001)
Hip Circumference	0.014^b^ (0.571)	HDL	-0.137^b^ (<0.001)
Waist Circumference	0.144^b^ (<0.001)	LDL	0.085^b^ (<0.001)
SBP	0.277^b^ (<0.001)	ALT	0.225^b^ (<0.001)
DBP	0.150^b^ (<0.001)	AST	0.182^b^ (<0.001)
FBG	0.185^b^ (<0.001)	CRP	-0.010^b^ (0.702)
HbA1c	0.309^b^ (<0.001)		

a Point biserial correlation.

b Spearman correlation coefficient, P-value in parenthesis.

To better understand the correlation between SOST and the other clinical covariates the data was stratified based on diabetes status and the correlations were estimated in the nondiabetic population **Table 3**. Within the non-diabetic patients, the point biserial correlation indicated that males tend to have significantly higher SOST compared to females. Furthermore, other covariates like age, HbA1c, SBP, ALT and AST showed higher and significant correlations compared to other covariates.

**Table 3:** Correlations between SOST and other covariates for non-diabetic patients (N=1425).

Covariate	SOST	Covariate	SOST
Gender^a^	0.250 (<0.001)	Insulin	0.041 (0.142)
Age	0.250 (<0.001)	TC	0.153 (<0.001)
BMI	0.015 (0.572)	TG	0.164 (<0.001)
Hip Circumference	-0.017 (0.545)	HDL	-0.105 (<0.001)
Waist Circumference	0.094 (<0.001)	LDL	0.142 (<0.001)
SBP	0.212 (<0.001)	ALT	0.198 (<0.001)
DBP	0.175 (<0.001)	AST	0.209 (<0.001)
FBG	0.071 (0.009)	CRP	-0.018 (0.523)
HbA1c	0.214 (<0.001)		

a point biserial correlation and others by Spearman correlation coefficient, P-value in parenthesis.

To quantify and test the effect of each covariate on the SOST, univariate and adjusted estimates were produced along with their 95% confidence intervals with quantile regression results presented in **Table 4**. Univariate quantile regression results indicated that gender, age, ethnicity, diabetes status, and HbA1c were independently and significantly associated with SOST as presented in **Table 4**.

**Table 4:** Associations between SOST and other demographic and clinical traits using quantile (median) regression analysis (N=2,083).

Covariate	Univariate Median Regression β (95%CI) (p-value)	Adjusted Median Regression β (95%CI) (p-value)
**Gender ** Female Male	** ** Ref 254.4 (217.8, 291.0) (<0.001)	** ** Ref 181.3 (140.8, 221.8) (<0.001)
**Age**, years	12.2 (9.8, 14.5) (<0.001)	8.8 (6.4, 11.3) (<0.001)
**Ethnicity ** Arab South Asian Southeast Asian	** ** Ref 237.4 (190.6, 284.2) (<0.001) 18.1 (-31.1, 67.4) (0.471)	** ** Ref 225.4 (169.7, 281.0) (<0.001) 121.9 (77.4, 166.4) (<0.001)
**Diabetes Status ** Non-Diabetic Diabetic	** ** Ref 297.7 (200.8, 394.7) (<0.001)	** ** Ref 91.5 (15.7, 167.3) (0.018)
**HbA1c**	98.3 (82.3, 114.2) (<0.001)	44.2 (10.5, 77.9) (0.010)
**BMI**	-1.08 (-4.0, 1.85) (0.469)	1.41 (-1.61, 4.42) (0.361)

Results from the adjusted model indicated that males have a higher median SOST by 176 units (96%CI: 137.3, 215.4) compared to females. For a one-unit increase in age, the median SOST increases by 8.9 units (95%CI: 6.8, 10.9). Compared to Arabs, South Asians have a higher median SOST of 224.1 units (95%CI: 169.7, 278.6) while Southeast Asians have a higher median SOST of 112.4 units (95%CI: 73.6, 151.1). Furthermore, patients with diabetes have a higher median SOST of 113.7 units (95%CI: 33.0, 194.4), while for a one-unit increase in HbA1c, the median SOST increases by 31.7 units (95%CI: 14.2, 49.2). Results showed that BMI was not significantly associated with SOST.

It is worth noting that when age was categorized using tertile values and BMI was categorized according to the WHO recommendations for obesity, the results were consistent with modeling age and BMI as continuous covariates which indicates the reliability of the results.

## 4 Discussion

This large cross-sectional study provides insight into the potential factors affecting circulating SOST levels in a multiethnic population. The data presented in the current study showed that diabetes status, HbA1c, age, gender, and ethnicity are the major factors associated with circulating SOST levels. This observation was further confirmed by quantile regression analysis where the associations were independent and remained significant even after adjusting for confounders. We also showed that obesity does not affect circulating SOST levels, as shown by the lack of correlation and the results of the quantile regression.

Patients with T2DM exhibited significantly higher SOST levels compared to their non-diabetic counterparts. Moreover, we observed strong correlations between SOST and HbA1c, FBG, and HOMA-IR, which are measures of long-term blood sugar control, blood sugar level, and insulin sensitivity, respectively. Among these variables, HbA1c showed the highest correlation with SOST, suggesting that SOST may reflect the chronic effects of hyperglycemia on bone health. Furthermore, the adjusted model highlighted the independent associations of SOST with diabetes and HbA1c. Our findings are in accord with previous reports (18, 19, 23, 28). In addition, Pacicca and colleagues conducted an *in vitro* study using the IDG-SW3 osteocyte-like cell line. When cultured under high glucose conditions, these cells exhibited a significant increase in SOST mRNA (by 100-fold) and SOST protein (by 5000-fold) compared to cells in control media. These findings indicate that glucose levels directly influence osteocyte function via SOST expression, shedding light on a potential mechanism by which high glucose/diabetes adversely affects bone quality. While the exact mechanism by which glucose homeostasis influences SOST levels is not fully understood, it is believed to involve the Wnt signaling pathway. A number of studies have demonstrated that activation of the Wnt signaling pathway enhances glucose metabolism (29, 30). Wnt7b has been shown to affect glucose uptake, GLUT expression, and Akt activation during osteoclastogenesis (31). Additionally, Wnt3a may boost mitochondrial oxygen consumption (32), elevate energy output by augmenting glutamine use in the TCA cycle (33), and activate the mTORC2 and protein kinase B (Akt) pathways, leading to the upregulation of essential glycolytic enzymes (34).

The age-associated increase of circulating levels of SOST in this study is in line with most previous investigations (35–37). Possibly, this is due to the natural aging process, which is known to affect bone turnover and remodeling (9, 38). SOST inhibits osteoblast activity, making it a potential biomarker for bone formation. The fact that the amount of SOST in our bodies increases as we age indicates that its increased production may reflect bone structure rather than size.

Moreover, there was a significant gender difference in median SOST levels, with males exhibiting higher levels compared to females. Gender disparities were observed across various factors, including diabetes status, insulin resistance (IR), body weight classifications, age brackets, and ethnicities (as illustrated in **Figures 3**, **4**). For example, a notable rise in SOST levels was evident among men compared to women within the diabetes cohort, a pattern consistent among non-diabetics, those with normal glucose tolerance, individuals with insulin resistance, and participants across different body weight categories (normal, overweight, obese), age groups, and ethnic backgrounds (Arab, South Asian, Southeast Asian). Our study findings underscore the significant role of gender in the variability of SOST levels. This observation aligns with previous studies that have reported gender-based differences in circulating SOST (36, 39, 40). Moreover, In a study conducted by MÖdder et al. involving 362 women and 318 men from a population-based cohort, it was observed that men consistently had higher serum SOST levels than women regardless of age (41). The exact reasons for this difference remain uncertain. However, one hypothesis the authors suggested was that the higher SOST levels in men could be attributed to their larger skeletal mass, which might produce and release more SOST into the bloodstream. In a second interventional study, the authors assessed the effect of estrogen on circulating SOST levels in both genders (37). They used two distinct direct intervention study methodologies (estrogen treatment and estrogen withdrawal) and found that estrogen treatment leads to a decrease in serum SOST levels, while estrogen withdrawal causes an increase in SOST levels. This evidence strongly suggests that estrogen has a regulatory effect on circulating SOST levels and, potentially, the production of SOST by osteocytes in the bone. Apparently, both hormonal and bone mass differences between genders might play a role in influencing circulating SOST levels. However, the exact mechanisms underlying these differences need further investigation.

The influence of ethnicity on SOST levels has not been comprehensively investigated. While several studies have examined the ethnic impact on SOST levels (23, 42, 43), there is still a need for a more detailed understanding. The study we present is one of the few that examines ethnic differences in the levels of circulating SOST. Our findings suggest distinct ethnic-specific variations in circulating SOST levels among Arabs, South Asians, and Southeast Asians. Notably, South Asian participants exhibited the most pronounced association, even when accounting for factors like age, gender, BMI, and diabetes status. This is in agreement with the observation of Janssen L. et al. who observed elevated plasma SOST levels in South Asians compared to white Caucasians (44). While their comparison was with an ethnic group different from the groups examined in our study, it underscores the unique metabolic challenges faced by the South Asian population (45, 46). Another study, focusing on 138 healthy pre-menopausal and post-menopausal women, included Chinese American and white Caucasian participants to identify ethnic variation in serum SOST levels (42). Their findings showed no significant ethnic-specific variations in SOST levels. However, they did note that post-menopausal women had higher SOST levels than pre-menopausal women across both racial categories. It is worth noting that the first important difference between our study and the other is that our study compared South Asians and Southeast Asians with Arabs rather than Caucasians; secondly, we included both genders, whereas their study included only women, and lastly, our larger sample size enhances the robustness of our results.

The relationship between obesity and circulating SOST levels is unclear in the literature. While some studies indicate a rise in serum SOST levels with obesity (35, 47, 48), others have not identified such a link (30, 43). In our study, we used three different anthropometric measurements to evaluate obesity, BMI, WC, and HC. We did not find any significant correlation between SOST and BMI or HC, but we found a weak correlation between SOST and WC. This suggests that obesity may not be a major determinant of SOST levels, and that other factors may have more influence. Our result is more reliable than previous studies, as we used a large sample size (n=2083) that increased the statistical power and reduced the sampling error.

Although our study controlled for age, gender, diabetes status, and BMI, we did not include assessments for Metabolic Associated Fatty Liver Disease (MAFLD). This omission limits our understanding of the full impact of metabolic comorbidities on SOST. Future research should consider incorporating a wider range of metabolic conditions, including MAFLD, to provide a more comprehensive view of how obesity and related disorders influence SOST levels. We also did not assess bone mineral density (BMD) in our study. We acknowledge the significance of BMD as a key marker of bone health, particularly in relation to sclerostin levels. Such data could enrich our research findings and implications. We hope to explore this in future work, including a comprehensive bone health assessment. We believe that our study’s findings lay the groundwork for such investigations.

In conclusion, the study confirms that circulating level of SOST is impacted by diabetes. The positive association between the level of SOST and various blood markers that are related to different metabolic complications implies that it plays a detrimental role in the individual’s wellbeing. As for the ethnicity, South Asian participants had the highest levels of SOST across the three groups and exhibited the most pronounced association, even when accounting for factors like age, gender, BMI, and diabetes status. This shows that the level of SOST may have a stronger impact on health conditions among people of specific ethnic background. This also sheds light on the importance of personalized medicine to address the development of medications taking into consideration the ethnic background.

## Data availability statement

The raw data supporting the conclusions of this article will be made available by the authors, without undue reservation.

## Ethics statement

The study protocol was approved by the Ethics Review Committee at Dasman Diabetes Institute under the reference RA2011-003, and done in accordance with the principles of the Declaration of Helsinki and good clinical practice guidelines. The studies were conducted in accordance with the local legislation and institutional requirements. The participants provided their written informed consent to participate in this study.

## Author contributions

TA: Writing – review & editing, Writing – original draft, Methodology, Investigation, Formal analysis. PC: Writing – review & editing, Methodology, Investigation, Data curation. IA-K: Writing – review & editing, Methodology, Investigation, Data curation. MA-F: Writing – review & editing, Writing – original draft, Supervision, Investigation, Formal analysis, Conceptualization. TT: Writing – review & editing, Methodology, Investigation, Formal analysis. AA: Writing – review & editing, Software, Methodology, Formal analysis. FS: Writing – review & editing, Methodology, Investigation, Formal analysis. HA: Writing – review & editing, Methodology, Investigation, Formal analysis. MA-G: Writing – review & editing, Methodology, Investigation, Formal analysis. JT: Writing – review & editing, Methodology, Investigation, Formal analysis. HK: Writing – review & editing, Methodology, Investigation, Formal analysis. FA-M: Writing – review & editing, Writing – original draft, Resources, Investigation, Formal analysis. JA: Writing – review & editing, Writing – original draft, Supervision, Methodology, Investigation, Formal analysis, Data curation, Conceptualization.
